# Genomic Predicted cross performance: a tool for optimizing parental combinations in breeding programs

**DOI:** 10.1093/database/baaf074

**Published:** 2025-11-17

**Authors:** Christine Nyaga, Marlee R Labroo, Agre Paterne, Asrat Asfaw, Marnin D Wolfe, Isaak Yosief Tecle, Lukas A Mueller

**Affiliations:** Section of Plant Breeding and Genetics, School of Intergrative Plant Sciences, Cornell University, 236 Tower Road, Ithaca, NY 14853, United States; Boyce Thompson Institute, 533 Tower Road, Ithaca, NY 14853, United States; Excellence in Breeding Platform, Consultative Group of International Agricultural Research, International Maize and Wheat Improvement Center (CIMMYT), Carretera México-Veracruz, Km. 45, El Batán, 56237, Texcoco, Mexico; International Institute of Tropical Agriculture (IITA), PMB 5320, Oyo Road, Ibadan Oyo State, 200001, Nigeria; International Institute of Tropical Agriculture (IITA), PMB 5320, Oyo Road, Ibadan Oyo State, 200001, Nigeria; Section of Plant Breeding and Genetics, School of Intergrative Plant Sciences, Cornell University, 236 Tower Road, Ithaca, NY 14853, United States; Boyce Thompson Institute, 533 Tower Road, Ithaca, NY 14853, United States; Section of Plant Breeding and Genetics, School of Intergrative Plant Sciences, Cornell University, 236 Tower Road, Ithaca, NY 14853, United States; Boyce Thompson Institute, 533 Tower Road, Ithaca, NY 14853, United States

## Abstract

**Background:**

Genomic prediction is an effective method for shortening breeding cycles and accelerating genetic gains. Traditionally, genomic prediction has focused on estimating ‘additive’ breeding values for individual genotypes. However, for many breeding programmes, predicting the cross-performance of parental combinations may provide greater value.

**Results:**

We present the genomic predicted cross-performance (GPCP) tool, which utilizes a mixed linear model based on additive and directional dominance. This tool is available within the BreedBase environment and as an R package. We assessed its effectiveness against classical genomic estimated breeding values (GEBVs) using simulated traits that exhibit varying dominance effects and on four yam traits. The GPCP tool proved superior to traditional methods for traits with significant dominance effects, effectively identifying optimal parental combinations and enhancing crossing strategies. This article outlines how the tool is implemented and emphasizes situations where predicting cross-performance is more advantageous than depending solely on GEBVs.

**Conclusions:**

The GPCP tool provides a robust solution for predicting cross-performance, offering significant advantages for breeding programmes targeting traits influenced by dominance. It is particularly useful for clonally propagated crops, where inbreeding depression and heterosis are prevalent and reciprocal recurrent selection is impractical.

## Introduction

Genomic prediction (GP) models have been used in plant and animal breeding to increase the selection accuracy of genotypes, reduce the costs of phenotyping, increase selection intensity, and decrease cycle length by recycling in earlier generations, leading to faster genetic gain [1, 2]. In rice, GP has been implemented to enhance selection for complex traits such as yield and disease resistance, with studies showing that prediction accuracies can accelerate breeding progress and shorten the development pipeline [3, 4]. In barley, GP has been applied across multi-environment trials to improve yield and malting quality traits, demonstrating its potential to optimize selection decisions in early generations and across diverse environments [5].

In recent years, several genomic estimated values such as genomic estimated breeding values (GEBVs) [6], genomic estimated general combining abilities (GEGCAs) [7, 8], genomic predicted cross performance (GPCP) [9], and others have been proposed as advantageous for given crop types, breeding schemes, and trait architectures. The appropriate genomic estimated value for use in breeding depends on whether there is appreciable inbreeding depression and heterosis in the trait index, the targeted breeding programme time horizon, and constraints of species reproductive biology. For example, breeding programmes with longer time horizons typically use a genomic estimated value that controls the inbreeding rate, such as the optimal cross value [10]. Programmes with appreciable trait inbreeding depression and heterosis may use GEGCA in a reciprocal recurrent selection programme rather than GEBV in a recurrent selection programme [11]. Programmes for which controlled crossing is difficult or impossible may avoid values such as GEGCA and GPCP in their traditional form, as they require controlled crossing [12, 13]. In clonal diploids, recurrent selection on GPCP is thought to be a useful strategy when substantial inbreeding depression and heterosis are present in a population trait index, but species biology or cost prevent the use of reciprocal recurrent selection on GEGCA [11–13].

In GPCP programmes, the cross-performance of parental combinations is predicted from parent marker genotypes and a training set of genotypes and phenotypes. The training model typically includes additive and directional dominance effects, although nearly any model can be used; with phenotypic selection, the expectation of cross performance is simply the average of the parental phenotypes, but this expectation can be refined with the availability of genomic information [14, 15]. The crosses with the highest predicted performance are selected to form the next breeding generation, where they may also be evaluated for release. The dual focus on additive breeding values and dominance deviation (DD) allows GPCP to maintain a higher proportion of dominance variance, particularly when inbreeding control is not imposed, than individual-based selection on GEBV alone [11]. GPCP can be further improved by adding a cross-usefulness criterion [13].

There are various approaches for GPCP. For example, Bernardo [16] primarily relies on simulation-based techniques, where potential progeny genotypes are simulated to evaluate both the mean and variance of cross outcomes. This approach enables the exploration of the distribution of genetic results through simulations. In contrast, Albrecht *et al*. [10] employ a formula-based method, predicting the mean genotypic value of a cross using equations that account for additive and dominance effects, without the need for simulations [17]. Jannink [18] builds on this by utilizing quantitative trait loci (QTL) results to predict both the mean and standard deviation of progeny, helping breeders better discriminate between crosses with high potential for superior individuals. Our method adheres to the formula-based approach, specifically utilizing the genomic predicted cross-performance (GPCP) model to estimate the mean genetic value of F1 progeny [15]. Experimental evaluations have shown that while GP models can predict progeny means with moderate to high accuracy, predicting family variance remains challenging, particularly for complex traits [15, 19]. These findings emphasize the limitations of prediction models in reliably forecasting genetic variability, important for selecting superior crosses. While dedicated tools such as COMA [20] and SimpleMating [21] have been developed, the practical implementation of GPCP within integrated breeding ecosystems is still lacking. The GPCP tool implements the formula-based approach in the widely-used BreedBase [22] environment allowing breeders to predict, save and manage crosses seamlessly. The GPCP tool is further implemented in CRAN R. It takes a dataset with genotypic information, linear selection index weights for traits, and fixed or random factors as inputs to be used. This study aims to (i) present the implementation of GPCP and (ii) evaluate its performance in enhancing genetic gain over cycles, especially for traits with significant dominance effects.

## Materials and methods

### GPCP on simulated dataset

The simulation study was conducted using the AlphaSimR package [23] to create four founder populations of *N* = (250, 500, 750, and 1000 individuals), each with 18 chromosomes (18 000 SNPs in total), and with chromosomes having 5400 segregating sites and 56 QTLs. The mean and additive variance were set to zero and one, respectively. To establish a realistic population structure with appropriate linkage disequilibrium and allele frequencies for the subsequent breeding programme simulations, a burn-in period of 10 generations was conducted. During this period, the initial founder populations underwent cycles of random mating with phenotypic evaluation.

Five uncorrelated trait scenarios were simulated with distinct DDs:

- Trait 1 was a purely additive trait set using addTraitA function in AlphaSimR thus had a mean DD of 0 representing a trait with negligible dominance effects. The narrow sense heritability was set to 0.6.
- Traits 2, 3, 4, and 5 representing non-negligible dominance effects that had a mean DD of 0.5, 1, 2, and 4, respectively, were set using the addTraitAD function. The narrow sense heritability was set to 0.3 for first three traits and 0.1 for trait 5.

This study modelled in one replication, a multi-stage clonal pipeline reflecting typical breeding practice. Each cycle proceeded through clonal evaluation (CE), preliminary yield trial (PYT), advanced yield trial (AYT), and uniform yield trial (UYT) before parent choice. At each stage, phenotypes were simulated with progressively higher heritability (CE: h² = 0.15; PYT: h² = 0.25; AYT: h² = 0.45; and UYT: h² = 0.65) and increasing replication (1, 2, 3, and 3 reps, respectively). Fixed proportions of individuals were advanced from one stage to the next (CE: 90%; PYT: 80%; AYT: 70%; and UYT: 60%), thereby mimicking attrition through the CE pipeline. Only the UYT pool was considered as the candidate parent set for GP. The GEBV approach selected parents based solely on additive marker effects, while GPCP selected parents based on their cross prediction merit. Both models were fitted using the sommer package [24], applying Best Linear Unbiased Predictions (BLUPs) with additive (and dominance, for GPCP) relationship matrices.

Each simulation ran for 40 cycles of selection after the burn-in, with both GEBV and GPCP methods applied at each population size and trait. At each cycle, top crosses (for GPCP) or top parents (for GEBV) were chosen to generate the next cycle of progeny, ensuring a consistent number of progeny across methods. The useful criterion (UC) and mean heterozygosity (H) were tracked per cycle to quantify genetic gain and diversity maintenance. The UC was calculated as the sum of mean genotypic value with the product of selection intensity at the cross level and standard deviation of the genetic value. The difference in UC (ΔUC = UC_GPCP_ − UC_GEBV​_) and heterozygosity (ΔH = H_GPCP_ − H_GEBV​_) across cycles were plotted in trend lines.

Factors varied included:

Population size: 250, 500, 750, and 1000 individuals
Dominance architecture: meanDD values of 0, 0.5, 1, 2, and 4, paired with heritability values (0.6, 0.3, 0.3, 0.3, and 0.1).
Number of crosses selected: a baseline B = 400 crosses plus three levels that is B + (initial population size divided by 2), B + (initial population size), B + (initial population size multiplied by 2).

### GPCP model

The GPCP model used is as presented by [14]


(1) \begin{eqnarray*}
y = X\beta {\mathrm{\,\,}} + f{b}{\mathrm{\,\,}} + {\mathrm{\,\,}}Z{a}{\mathrm{\,\,}} + W{d}{\mathrm{*\,\,}} + {\mathrm{\,\,}}{e},
\end{eqnarray*}

where $y\,\,$is a vector of phenotype means, $X$ is an incidence matrix, and $\beta $ represents the vector with the fixed effects estimated in the model. $f{\rm b}$ models directional dominance, where $f$ represents the vector with inbreeding coefficients and $b$ a parameter indicating the effect of genomic inbreeding on performance. $a$ is a vector of the additive effects, and $d*$ a vector of the dominance effects not captured by $fb$. The *Z* matrix stores allele dosages; for diploids these values are 0, 1, 2, while for tetraploids and hexaploids they are 0…4 or 0…6, respectively. These allele dosages scale the additive effects vector in the prediction model. The *W* matrix captures heterozygosity; in diploids it is coded 0 for homozygous genotypes and 1 for heterozygotes, and for higher ploidies it represents the proportion of heterozygous allele combinations. Lastly $e\,\,$is a vector of residual effects. Random effects $a$, $d*_{}^{}$, and $e$ were assumed to be normally distributed with mean zero and variance $\sigma _a^2$, $\sigma _{d*}^2$, and $\sigma _e^2$, respectively.

Parent selection is based on the GPCP method that predicts the mean genetic value of the F1 progeny by incorporating both additive and dominance effects of SNP markers, following the approach described by [11]. This method focuses on parent complementarity and directly accounts for the predicted amount of heterosis in the selection process. The prediction is based on the differences in allele frequencies between the two parents, which allows for the maximization of the mean genotypic value in the progeny. Therefore, the cross-performance prediction is centered on the mean of the F1, without modelling the variance or segregation density.

In our method, the mean genotypic value of the F1 progeny (${M_{F1}}$) is predicted using the following equation, which sums the additive and dominance effects across SNP markers:


(2) \begin{eqnarray*}
{M_{F1}}\,\, = \,\,\mathop \sum \limits_{i = 1}^n \left[ {{a_i}\left( {{p_i}\,\, - \,\,{q_i}\,\, - \,\,{y_i}} \right)\,\, + \,\,{d_i}\left( {2{p_i}{q_i}\,\, + \,\,{y_i}\left( {{p_i} - {q_i}} \right)} \right)} \right]. \\
\end{eqnarray*}

In this equation, ${a_i}$​ and ${d_i}$ represent the additive and dominance effects for the $i$th SNP, ${y_i}$ is the difference in allele frequency between the two parents at the *i*th locus, calculated as ${y_i}$ = ${p_i}$−$p{^{\prime}_i}$ = ${q_i}$−$q{^{\prime}_i}\,\,$, where ${p_i}$ and ${q_i}$ are the allele dosages in one parent, and $p{^{\prime}_i}$ and $q{^{\prime}_i}$ are the allele dosages in the other parent and applies unchanged to polyploid parents.

This approach allows direct incorporation of heterosis through parent complementarity, which is captured by the differences in allele frequencies between the parents. By doing so, we can predict the mean performance of the cross, focusing specifically on maximizing the average genetic value in the progeny.

### GPCP validation using yam dataset

In addition to the simulation study, GPCP was validated using real yam breeding data from the 2020 to 2022 breeding cycles. The dataset comprised 52 full-sib families with a total of 2302 progenies generated at the seed progeny stage in 2020. These progenies were multiplied to produce 29 165 plantlets, averaging 13 plantlets per progeny (range: 1–38; median: 10). At the tuber progeny stage in 2021, all progenies were evaluated in a partially replicated trial at first clonal generation for key agronomic traits, including yield per plant (Yield), tuber length (Tlength), average tuber weight (ATW), and a disease trait yam mosaic virus (YMV). Genotyping was performed using DArTSeqLD. GPCP was applied at the clonal nursery stage in 2022, selected clones were advanced, and new crosses were made based on predicted cross merit. Two selection classes were defined based on cross-prediction merit scores:

Class 1: Crosses with merit > 3 (*n* > 1 500 combinations), with 325 unique parents and 72 phenotypically selected clones.
Class 2: Crosses with merit between 2 and 3 (*n* > 55 000 combinations), with 1639 unique parents and 86 phenotypically selected clones.

A total of 158 clones were selected, corresponding to a 6.86% selection intensity, effectively reducing the breeding cycle to 3 years.

## Results

### Random mating during burn-in phase ensured an unbiased baseline

**Figure 1.**
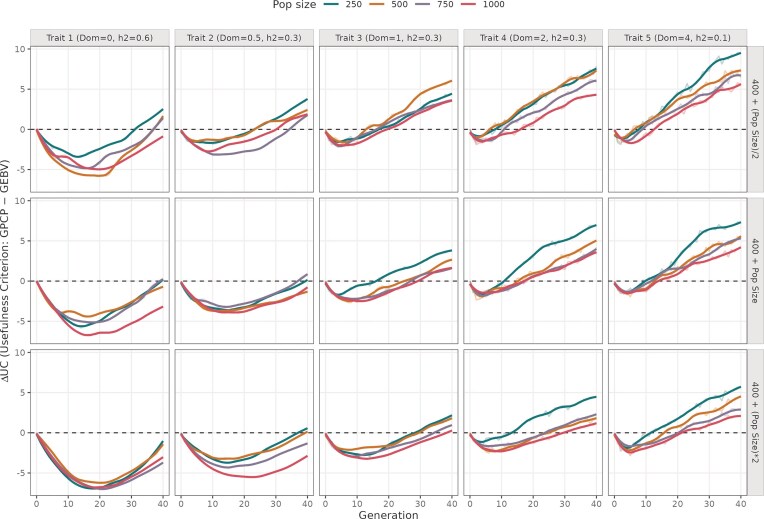
Difference in UC values from using GPCP and GEBV models across 40 breeding cycles. The plot is divided into five columns showing simulations run with different mean DD values (0, 0.5, 1, 2, and 4), and three rows indicating different number of advanced crosses. The colored trend lines indicate different initial population sizes ranging from 250 to 1000 individuals.

During the 10-cycle burn-in (cycles −9–0), UC hovered around zero for every scenario, confirming that mating was effectively random and that no unintended directional selection crept in before the experimental phase began (Fig. S1). This phase produced the intended baseline of linkage disequilibrium and allele frequencies before selection began. The sharp change at Cycle 1 therefore isolates response attributable to the selection models (Fig. 1).

### GPCP outperforms GEBV for long-term genetic gains with increasing dominance, fewer crosses, and smaller breeding programmes

In the majority of the panels (Fig. 1), GEBV model tends to maximize short-term genetic gains, whereas GPCP excels in achieving long-term genetic gains. In the early cycles, ΔUC was negative, reaching a minimum between cycles 5 and 25, before gradually rising towards zero and becoming positive by cycle 40. This trend indicates that GPCP’s advantage becomes clearer in later cycles, especially under conditions that shape long-term genetic gain.

For traits with purely additive effects (Dom = 0), the difference in genetic gain between GPCP and GEBV (∆UC) reaches its minimum most slowly compared to traits with dominance, showing only marginally positive values in later cycles (cycles 30–40). However, as DD increases to partial dominance (Dom = 0.5), complete dominance (Dom = 1), and overdominance (Dom > 1), the minimum ∆UC value becomes more pronounced, and late-cycle ∆UC increases significantly. For example, with partial dominance, ∆UC remains below 5, whereas for a trait with overdominance, ∆UC ranges between 5 and 10 in the top row of Fig. 1. This shows that GPCP increasingly outperforms GEBV as dominance effects strengthen.

This advantage of GPCP is further amplified when fewer crosses are advanced (e.g. nCrosses = B + ½ initial population size), where ∆UC is largest, decreasing as more crosses are included. Similarly, smaller initial population sizes, such as 250 individuals, enhance GPCP’s performance, with ∆UC recovering earlier and reaching higher positive values compared to larger populations of 750 or 1 000 individuals. These trends highlight GPCP’s superior ability to sustain long-term genetic gains under conditions of increased dominance, limited crosses, and smaller breeding programmes.

### GPCP better maintains heterozygosity across all panels

**Figure 2.**
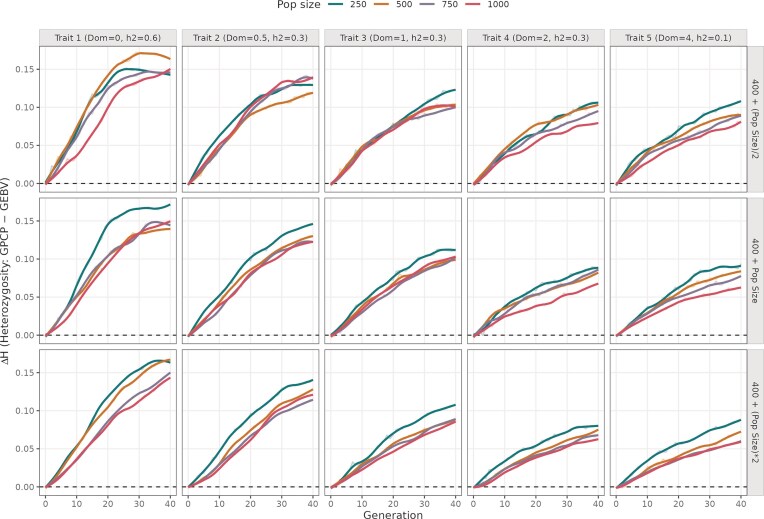
Difference in mean heterozygosity values from using GPCP and GEBV models across 40 breeding cycles. The plot is divided into five columns showing simulations run with different mean DD values (0, 0.5, 1, 2, and 4), and three rows indicating different number of advanced crosses. The colored trend lines indicate different initial population sizes ranging from 250 to 1000 individuals.

In every panel, ΔUC is positive from the early cycles and rises as selection proceeds, indicating that GPCP consistently maintains more heterozygosity than GEBV (Fig. 2). This increase is the steepest during the first ∼20 cycles and then plateaus to ΔH values between 0.05 and 0.2. The ΔH was highest mostly in programmes with fewer crosses, lower dominance and smaller initial population sizes.

### GPCP identifies yam progenies with superior performance in agronomic and disease traits

**Figure 3.**
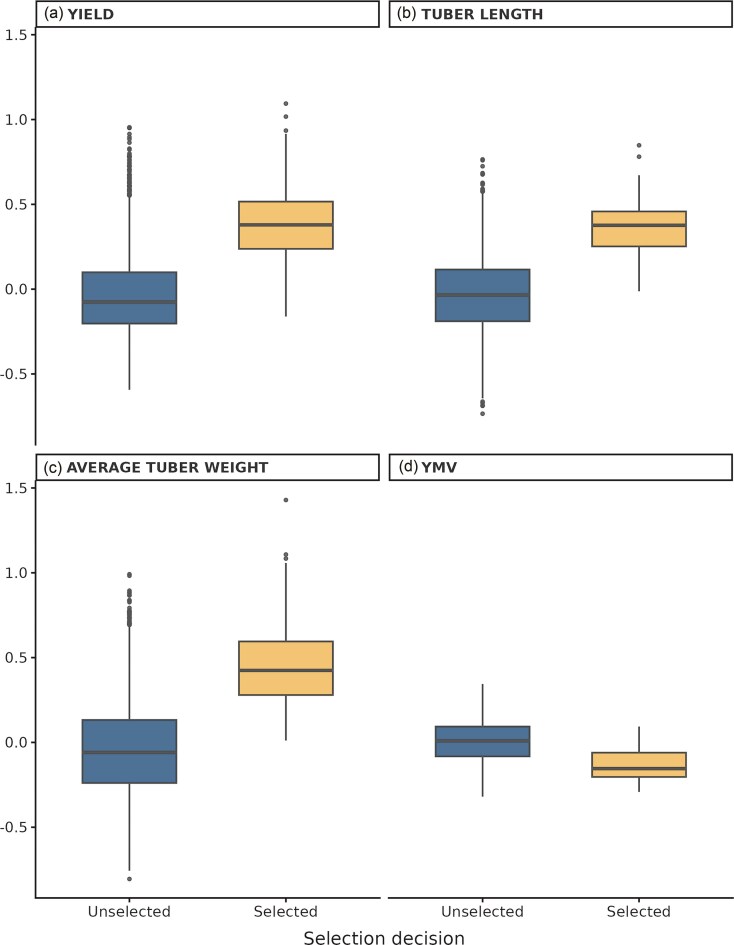
Trait performance of selected and unselected yam progeny using GPCP. Boxplots show standardized values for (a) yield per plant (Yield), (b) tuber length, (c) ATW, and (d) YMV. Selection decisions (selected vs. unselecteD) were compared using Wilcoxon rank-sum tests, with all traits showing significantly higher median values in the selected group (*P* < 2.22 × 10^−16^).

Across all evaluated traits, selected progenies had higher median values than unselected progenies (Fig. 3). For ATW, the median value for selected progenies was ∼0.42, compared with −0.06 for unselected progenies (Wilcoxon test, *P* < 2.22 × 10^−16^). For tuber length (Tlength), the median value for selected progenies was ∼0.38, compared with −0.03 for unselected progenies. For yield per plant, the median value for selected progenies was ∼0.38, while the median for unselected progenies was −0.08. Lastly for disease trait YMV, the median of selected progenies was 0.38 while unselected was 0.66 indicating superior performance for selected progenies since with disease traits, lower values indicate higher resistance to disease.

## Discussion

Simulations provide an effective method for analysing the key elements in breeding programmes, which often span many years. This approach allows for the controlled manipulation of features, enabling rapid, cost-effective, and consistent inferences on genetic parameters across multiple cycles [25]. The present study assessed the performance of GPCP relative to GEBVs within GP frameworks using simulated and real datasets. GEBV selects parents based on additive effects; the sum of the predicted average effects of marker alleles of an individual [26], while GPCP predicts the mean performance of the F1 family by including both additive and dominance marker effects and prioritises complementary allele frequencies between parents [27].

The results of the current study showed that GEBV provided higher genetic gain than GPCP in earlier cycles indicating that it prioritized short-term gains while GPCP had higher gains later on. This is because GEBV truncation focuses on individual merits [26] thus excelling in the beginning, while GPCP invests in crosses expected to produce superior progeny after segregation within families [27]. As recombination reshapes linkage disequilibrium and within-family selection samples the upper tail of families with large segregation variance, the advantage predicted by the usefulness criterion becomes visible and ultimately exceeds that of GEBV, especially when dominance or overdominance contributes to performance [28].

This study simulated a purely additive trait that mimics dry matter content in cassava [29, 30]. Consistent with this, other studies have reported little to no improvements in short-term prediction accuracy and genetic gain when modelling dominance for traits with low dominance ratios [9, 29, 31]. Conversely, with dominance modelled after yield related traits [29, 30, 32], GPCP achieved higher genetic gain than GEBV and the advantage increased with the dominance ratio. This pattern mirrors earlier findings that dominance and other non–additive effects strongly influence hybrid performance and that including dominance effects in genomic models yields higher prediction accuracy than additive–only models when dominance effects are high [27, 29–31, 33]. The greater performance of GPCP at higher dominance values arises because the model directly captures heterosis by summing dominance marker effects and by selecting complementary allele frequencies exploiting DDs in the F1, producing superior crosses. When comparing the two models across different dominance levels, incorporating dominance effects can be crucial for reducing bias from additive variance, thereby preventing underfitting. These findings reinforce that selecting an appropriate model is highly dependent on the underlying trait architecture [34].

Programme design particularly the number of crosses advanced and initial population sizes affect the magnitude of genetic gain realized. When few crosses advance, each mating decision has higher leverage, therefore a method (GPCP) that recognizes within-family variance yields higher genetic gain. As the number of crosses increases without increase in distinct parents, programmes begin to recycle the same alleles and the marginal benefit of additional crosses diminishes with both strategies sampling from a similar mating space and converge in outcomes [10]. Smaller initial candidate pools in this study magnified GPCP’s advantage. Previous studies attribute this to effective population sizes whereby in small programmes, co-ancestry rises quickly under truncation. Methods such as GPCP that spreads contributions across more parents and prioritizes families with usable segregational variance produces higher gains and retains more diversity [10].

Maintaining genetic diversity is crucial for sustaining long–term genetic gain [35]. Rapid depletion of diversity can reduce the genetic variance available for future selection and increase inbreeding depression. Genetic diversity, quantified here as mean heterozygosity, decreased over the selection cycles for both GEBV and GPCP, as expected under directional selection; however, GPCP maintained more heterozygosity than GEBV showing that it balances well genetic gain with genetic diversity. This is consistent with long–term genomic selection simulations have shown that accumulated heterozygosity decreases more slowly when non–additive effects are present [34], and that larger populations or optimal cross selection strategies can reduce the rate of heterozygosity loss [35]. When only additive effects are considered, selection tends to favour individuals with the highest allele substitution values, which can quickly fix favourable alleles and deplete genetic variability [36]. By incorporating dominance, however, the prediction accounts for complementary allele interactions between parents, rather than only their average additive values. This means crosses are prioritized not solely for immediate gain but also for their ability to exploit heterozygosity, buffering against the rapid erosion of diversity [13].

Overall, the simulation findings support the potential advantages of GPCP over GEBV in breeding programmes targeting traits with significant dominance effects. By achieving higher genetic gains and maintaining greater genetic diversity, GPCP may offer a more effective approach for long-term genetic improvement.

GPCP also demonstrated practical effectiveness in real breeding context as shown in the yam dataset whereby the selected crosses had significantly higher performance than the unselected across all the traits. Previously, Adejumobi *et al*. [37] applied GPCP to phenotypic and genotypic data from yam, successfully identifying high-merit parental combinations for breeding. Similar validations have been reported in cassava [13] and soybean [38] where predicted cross performance aligned with field outcomes. These studies confirm that insights from simulations translate to operational breeding programmes. Future research, however, should focus on exploring the integration of additional genetic effects, such as epistasis, into the GPCP framework. Additionally, expanding the GPCP model to accommodate a wider range of trait architectures and breeding strategies could further enhance its applicability and effectiveness [17, 39, 40].

## Implementation of GPCP as a CRAN R package

To utilize the gpcp package developed in R, begin by installing the necessary dependencies. First, install BiocManager to manage bioinformatics packages. Next, install VariantAnnotation and snpStats via BiocManager:: install(). Install sommer if not already installed using install.packages(‘sommer’).

Once these dependencies are in place, the gpcp package can be installed using install.packages(‘gpcp’). After installation, load the phenotype data from a CSV file and specify the genotype file, which can be in either VCF or HapMap format. Define essential inputs, including the column name for genotype IDs, traits to predict, weights for each trait, fixed effects, ploidy level, and the number of crosses to predict. In the example below, we use the diploid ploidy level case. Finally, execute the runGPCP function to generate the predicted cross performance, which can be reviewed using the head() function for further analysis.

In the code snippet below, a yam dataset is used for illustration purposes in the case of diploid level. This dataset includes genotypic data with ploidy level of 2 and phenotypic data whereby Yield and Dry Matter Content (DMC) are used with selection indices (weights) as 3 and 1, respectively. The number of crosses selected is set as 150. These parameters are then passed into the function runGPCP and finally the first top crosses are viewed using the R head function.

Another polyploid dataset obtained from sommer R package is used for the polyploid level case whereby the Ploidy level is set to 4.

**Table:**
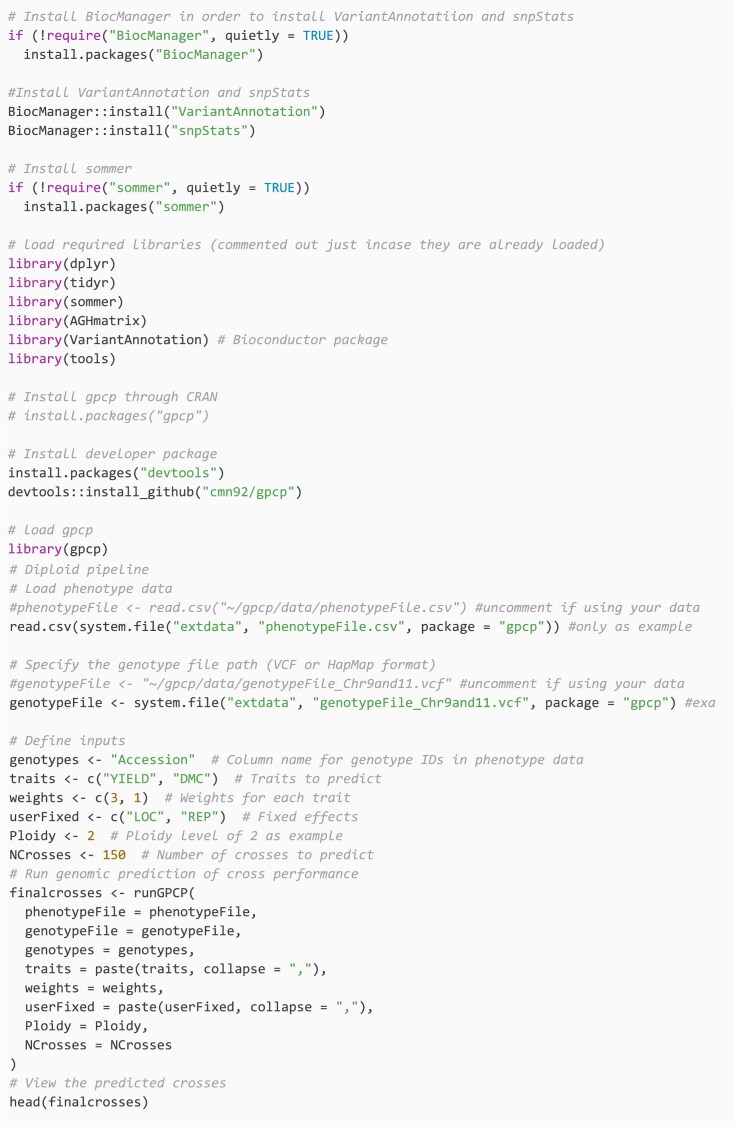

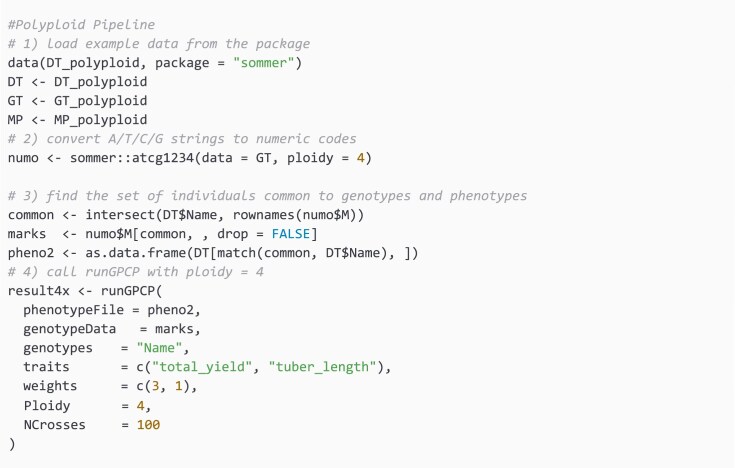


The function routine scales linearly, *O*(*m*), with the number of markers *m*, but grows quadratically, *O(n^2^)*, in the number of individuals *n* because it exhaustively computes expected performance for all *n*(*n* − 1)/2​ pairwise crosses (Figs S2 and S3).

## Implementation of GPCP on Breedbase

**Figure 4.**
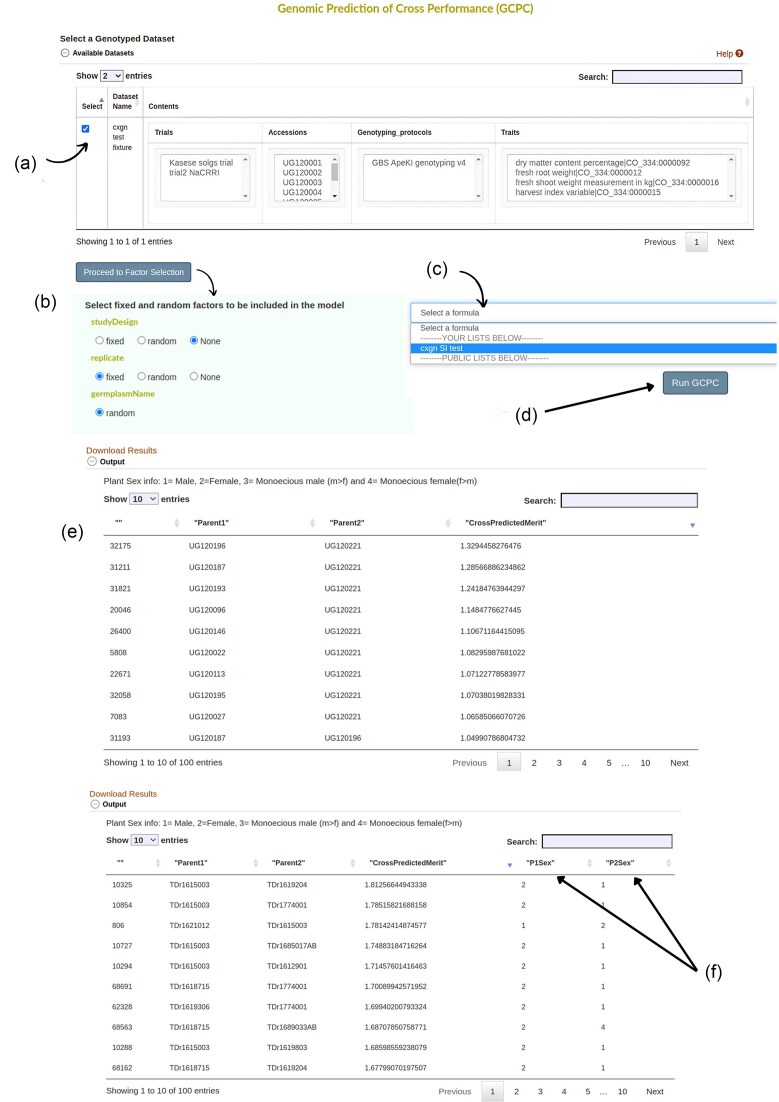
The input page of the GPCP tool showing different user interface elements. (a) Available datasets checkmarked to show the selected dataset for further analysis. (b) Available factors are loaded once ‘Proceed to Factor Selection’ is clicked. Options to choose from include ‘fixed’, ‘random’, and ‘None’. (c) A dropdown menu with previously created formulas for selection indices. (d) Once all inputs have been selected, ‘Run GPCP’ button prompts the system to run the model. (e) shows the final results of the top 100 crosses ordered in descending order based on cross prediction merit. Results can be downloaded by clicking on ‘Download Results’. (f) The model includes plant sex information if available and outputs it on the Table, otherwise, it follows the output given by (e). As indicated 1 = Male, 2 = Female, 3 = Monoecious male (m > f), and 4 = Monoecious female (f > m).

In Breedbase, input data for an analysis is selected by using the ‘dataset’ concept [22]. A worked illustration using yam as a case crop to predict cross-performance using the tool is detailed as follows. First, a yam dataset containing the individuals with their genotype and phenotype data was created using the Search Wizard (Fig. S4). In the yam dataset, two trials Kasese and NACCRI were used with the genotyping protocol as GBS version four. Second, a selection index with traits of interest was created using the Selection Index tool. The traits used were dry matter content, fresh shoot weight, fresh root weight, and harvestable index with selection indices (0.5, 1, 1, 0.5, respectively). Third, the GPCP tool is selected from the Analyze menu. Then, the yam dataset created with the desired genotyping protocol and phenotypic information was chosen from the dataset selector on the page (Fig. 4a). Clicking on ‘Proceed to Factor Selection’ loads the available factors that can be included in the model.

The factors to be included in the model were fitted either as Fixed or Random. In this case, studyDesign was set as None and replicate as a fixed factor. Click ‘None’ for factors that should not be included in the model (Fig. 4b). Note that the ‘germplasmName’ is always factored as Random, and this setting can’t be changed. The next step is to select the selection index for your traits on the dropdown menu whereby ‘cxgn SI test’ was selected (Fig. 4c). Clicking ‘Run GPCP’ then runs the model (Fig. 4d). The output is presented in the form of a Table with ‘ID’, ‘Parent1’, ‘Parent2’ and their cross-prediction merit organized in descending order (Fig. 4e).

For dioecious plants, such as yam (*Dioscorea spp*.), the results will also have sex information if the dataset has plant sexes available in the database (Fig. 4f).

In conclusion, we developed a new web tool for predicting cross performance using genomic data. GPCP exploits both additive and directional dominance thereby increasing the heterozygosity level relative to selection on GEBV with random mating and is useful for clonally propagated crops where inbreeding depression and heterosis are substantial.

## Conclusion

In conclusion, GPCP demonstrates promising performance compared to GEBV, particularly for traits influenced by dominance. By facilitating higher genetic gains and better preservation of genetic diversity, GPCP has the potential to contribute to more efficient and sustainable breeding programmes. Continued advancements in GP models and computational tools will be essential for realizing the full benefits of GPCP in applied breeding contexts. Future advancements in the GPCP tool will include incorporation of the cross merit variance in the CRAN and BreedBase environments.

## Supplementary Material

baaf074_Supplemental_Files

## Data Availability

Project name: Genomic Predicted Cross Performance, GPCP. Project home page: https://www.cassavabase.org/tools/gcpc (username- gpcp_reviewer, password- predict_combinations) https://github.com/solgenomics/sgn CRAN R package: https://cloud.r-project.org/web/packages/gpcp/index.html, Developer R package on github https://github.com/cmn92/gpcp. The data underlying this article are available in github at https://github.com/cmn92/GPCP-PAPER.git. and can be accessed with "cmn92/GPCP-PAPER".
