# Supplementary figures and images for: Genomic Predicted cross performance: a tool for optimizing parental combinations in breeding programs
Source: Database (Oxford). 2025 Nov 17;2025:baaf074. doi: 10.1093/database/baaf074 (PMC12620651; doi:10.1093/database/baaf074)

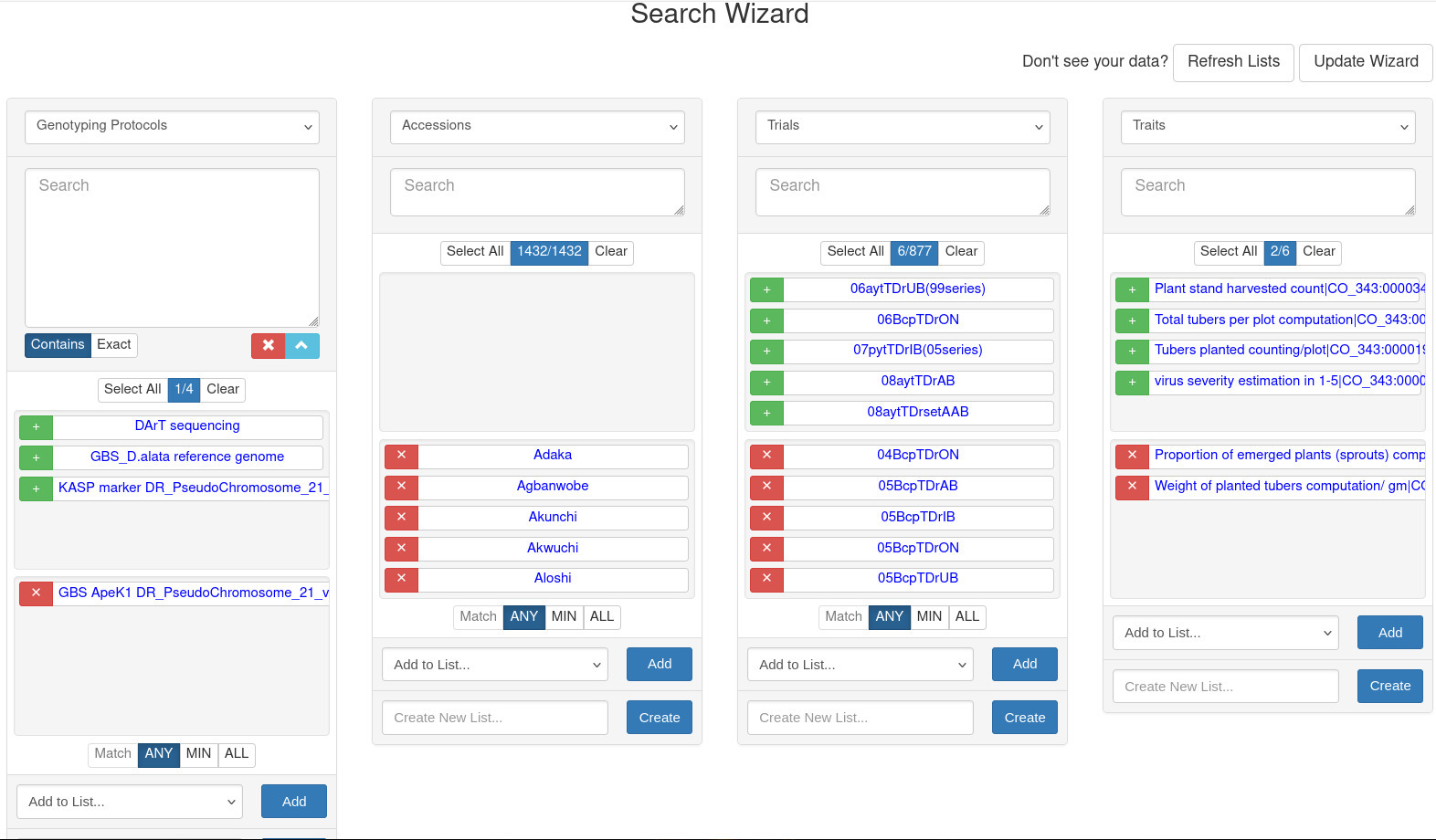

Supplement: baaf074_Supplemental_Files [file baaf074_supplemental_files.zip › Supplementary Figure 4 searchwizard.jpg]

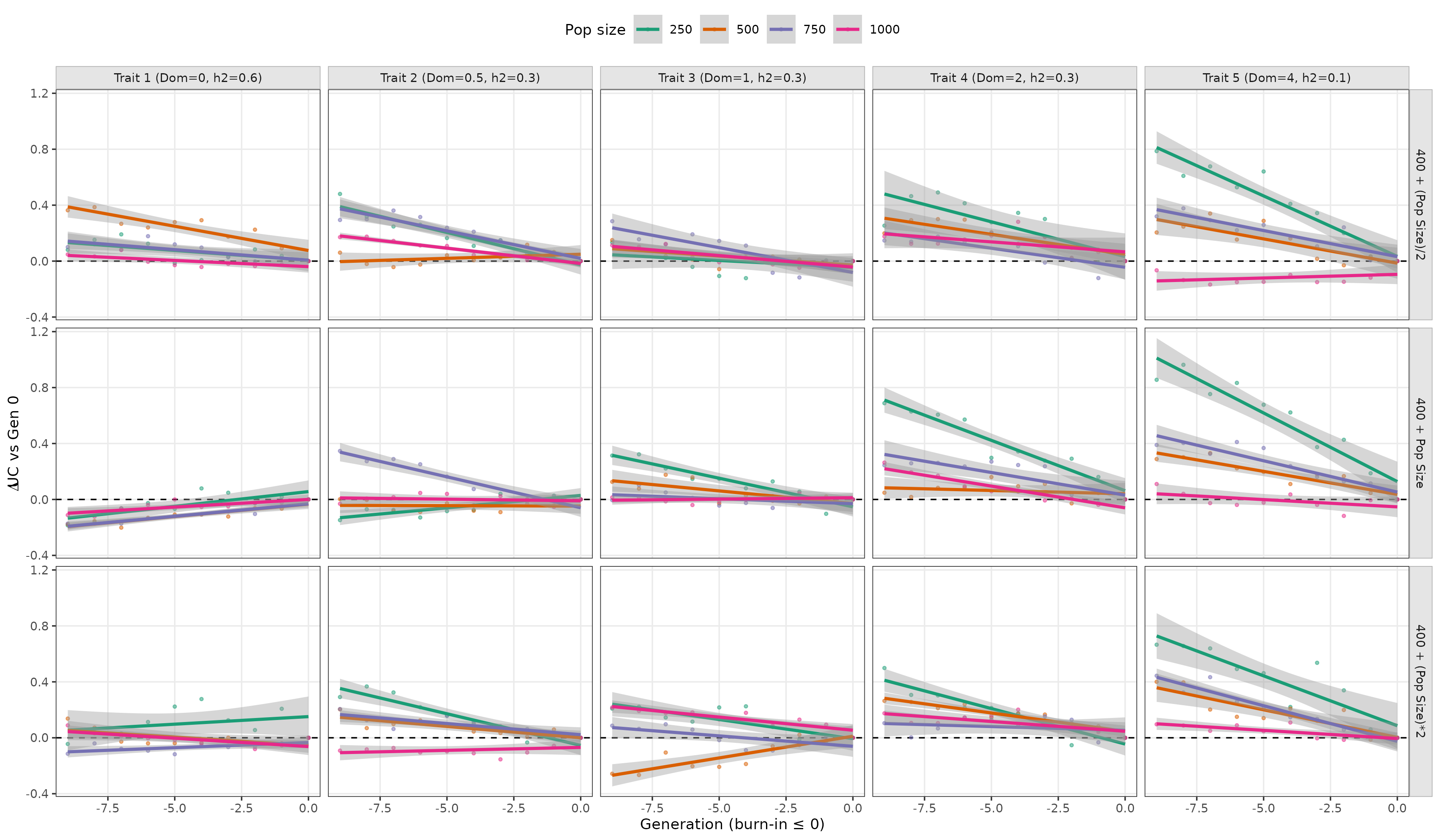

Supplement: baaf074_Supplemental_Files [file baaf074_supplemental_files.zip › Supplimentary Figure 1 _burnin_delta_UC_CMYK.tif]

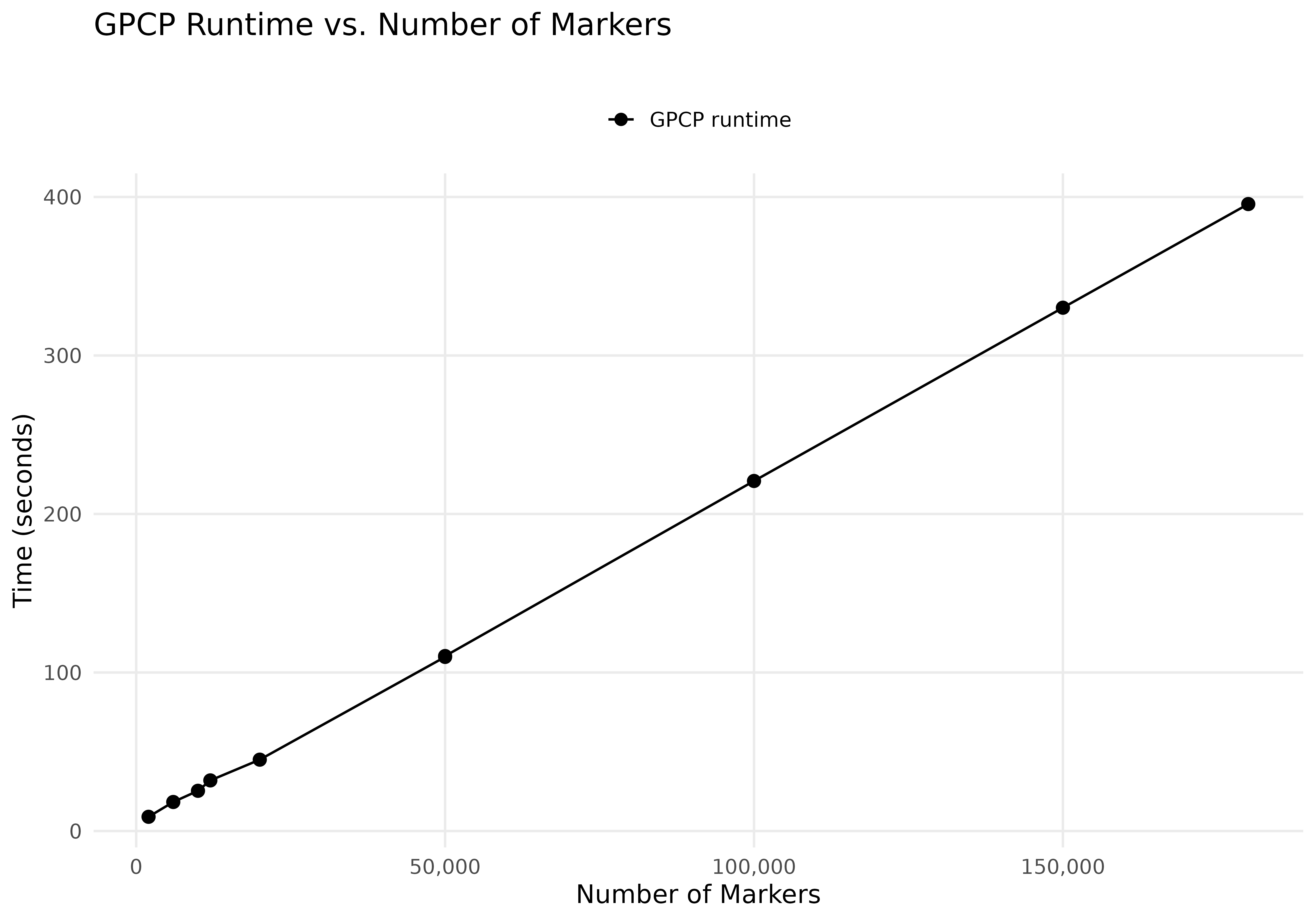

Supplement: baaf074_Supplemental_Files [file baaf074_supplemental_files.zip › Supplimentary Figure 2_runtime_markers_CMYK.tif]

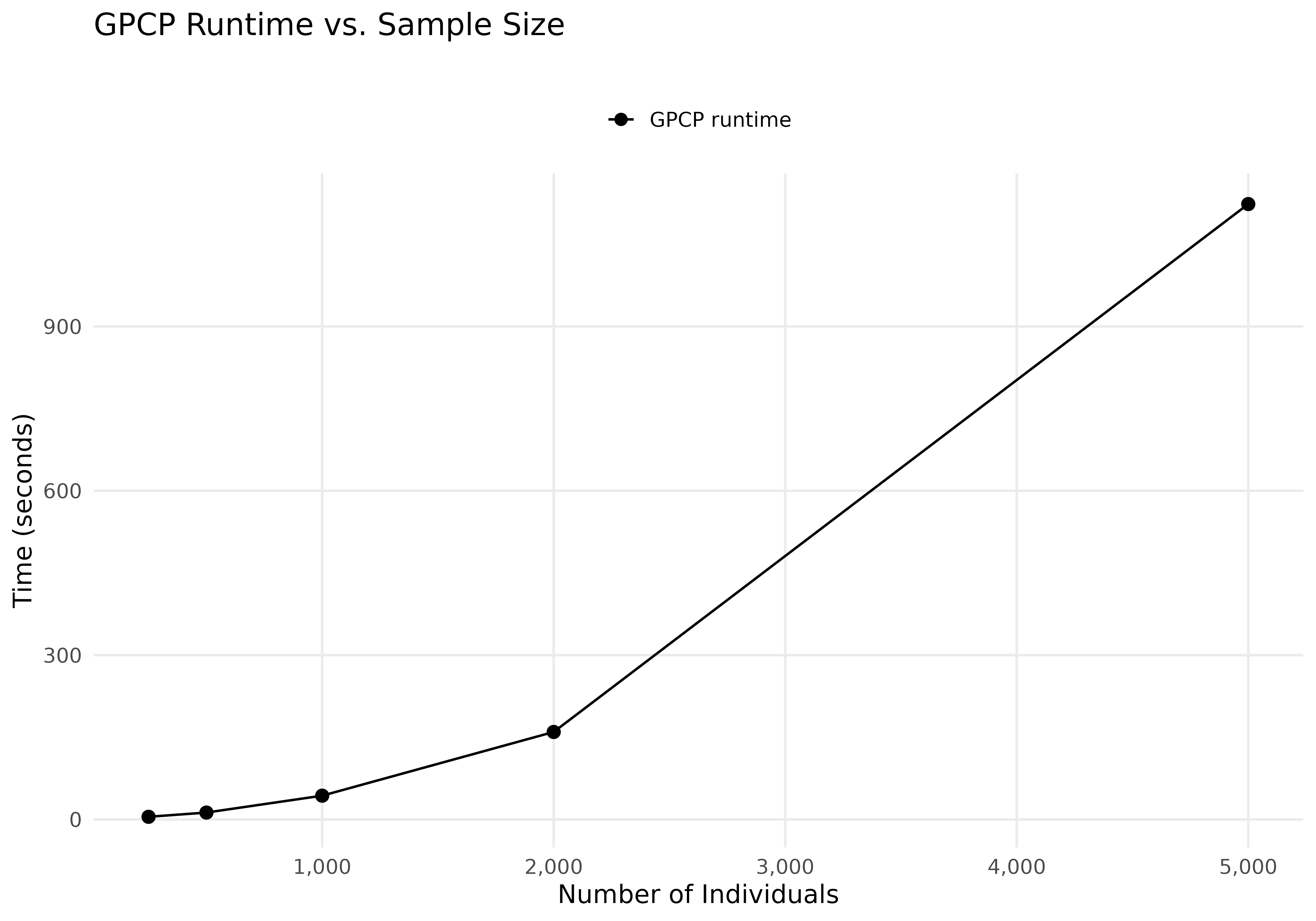

Supplement: baaf074_Supplemental_Files [file baaf074_supplemental_files.zip › Supplimentary Figure 3 _runtime_individuals_CMYK.tif]
